# Are all children treated equally? Psychiatric care and treatment receipt among migrant, descendant and majority Swedish children: a register-based study

**DOI:** 10.1017/S2045796022000142

**Published:** 2022-04-19

**Authors:** Ester Gubi, Hugo Sjöqvist, Christina Dalman, Sofie Bäärnhielm, Anna-Clara Hollander

**Affiliations:** 1 Global Public Health, Karolinska Institute, Stockholm, Sweden; 2 Transcultural Center, Stockholm, Sweden

**Keywords:** Child psychiatry, epidemiology, mental health, multicultural, psychiatric services

## Abstract

**Aims:**

Underutilisation of mental health services among migrant youth has been demonstrated repeatedly, but little is known about potential discrepancies in terms of treatment receipt for those who do reach services. This study examines the type and level of care received among migrant children and descendants of migrants, particularly investigating disparities in treatment receipt given a specific diagnosis.

**Methods:**

We used register data of the total population aged 6–17 years in Stockholm, followed from 2006 to 2015, comprising 444 196 individuals, categorised as refugees, non-refugee migrants, descendants of migrants and Swedish-born. To identify recommended treatments for specific diagnoses we used official clinical guidelines. We report logistic regression estimated odds ratios (ORs) and 95% confidence intervals (CIs) of diagnosis receipt, treatment provision and level of care where a diagnosis was first registered.

**Results:**

Migrant children had a lower likelihood of receiving a wide range of psychiatric diagnoses, including mood disorder (OR 0.58; 95% CI 0.52–0.64), anxiety disorder (OR 0.62; 95% CI 0.57–69) and neurodevelopmental disorder (OR 0.59; 95% CI 0.55–0.63). Moreover, when these diagnoses were set, migrant children had a lower likelihood of receiving the recommended treatments for these conditions compared to the majority individuals with the same diagnosis (OR of receiving psychotherapy for anxiety disorder and depression: 0.71; 95% CI 0.62–0.95 and 0.50; 95% CI 0.33–0.75, respectively; OR for receiving ADHD-medication: 0.49; 95% CI 0.43–0.54).

**Conclusions:**

Migrant children risk underdiagnosis of various mental health conditions, and, when reaching mental health services, risk not receiving the optimal care available.

## Introduction

Mental and substance use disorders are the leading cause of disability among children and youth in high-income countries (Erskine *et al*., 2015) and extensive evidence shows that resettled refugee children face greater risks of mental illness compared to their majority peers (Bronstein and Montgomery, 2011). Non-refugee migrant children and descendants of migrants may also be at increased risk of poor mental health (Carlerby *et al*., 2011; Curtis *et al*., 2018). Despite this, lower and unfavourable use of psychiatric care among migrant youth compared to their majority peers has been demonstrated repeatedly (Colucci *et al*., 2014; de Montgomery *et al*., 2020). Moreover, findings suggest that certain diagnoses may be underdiagnosed among migrant groups (Morinaga *et al*., 2020), and structural and informal barriers may impede the receipt of the most optimal care available (Norredam *et al*., 2007). Thus, the problems are complex: not all who need care reach care; those who reach care may be misdiagnosed, and little is known whether, among those who receive a diagnosis, the recommended treatments are in fact provided. To the best of our knowledge, this is the first study to examine potential differences between majority and migrant children who are in psychiatric care and have received the same psychiatric diagnosis in terms of specific treatment provision.

Our research questions were the following: What are the differences between migrants and descendants, as compared to Swedish majority children, in terms of receiving a specific psychiatric diagnosis, level of care where the diagnosis is set, and of receiving clinically recommended treatments given a specific diagnosis? Sweden has a universal welfare system that guarantees all children regardless of residency status the right to care and the right to free-of-charge language interpreters. Nonetheless, studies show discrepancies in psychiatric care utilisation among migrants and the majority minors in Sweden (Berg *et al*., 2020) and factors such as time in Sweden and reason for migration have been shown to affect utilisation patterns. Although increased residency has been shown to increase care use (Brendler-Lindqvist *et al*., 2014; Manhica *et al*., 2017; Berg *et al*., 2020), the pattern is not completely consistent, and young refugees have been shown to use more care than their non-refugee migrant counterparts early upon arrival (Gubi *et al*., 2022). Moreover, while low household income increases the risk for psychiatric utilisation in general, findings regarding the effect of parental SES on migrant children's mental health service use have been inconclusive, highlighting the need for further investigation (Barghadouch *et al*., 2016; Finnvold 2019; de Montgomery *et al*., 2020).

Sweden has a high standard of official population registers adapted for psychiatric research, and Stockholm, the capital, has high-quality data on psychiatric care utilisation on a very detailed level which, combined with the fact that Sweden has been one of the most accepting countries in the European Union in terms of immigration, makes it an excellent setting in which to conduct this research. We hypothesise that migrants and descendants have a lower risk of receiving certain types of diagnoses and higher risk for others; more often be diagnosed in-patient and primary care compared to Swedish majority children; receive fewer psychotherapy-based treatments, and for certain conditions such as ADHD, fewer psychotropic drugs, compared to Swedish majority children; and that parental time in Sweden, parental socio-economic status and reason for migration would explain some of the differences we expect to find.

## Methods

### Data sources

Data were extracted from a longitudinal database of linked national and regional registers, called Psychiatry Sweden (held by our research group), which includes all Swedish residents since 1932, linked by a unique personal identification number and anonymised by Statistics Sweden for research purposes. We obtained all exposure, outcome and covariate data from this linkage (see Forslund *et al*., 2020) for details about the database and the component registers.

### Study population

The study population consisted of all children aged 6–17 years living in the Stockholm Region between 1 January 2006 and 31 December 2015, making up a total of 487 065 individuals. We excluded adopted individuals (*n* = 4699), and those with missing data on parental country of origin or missing parental income data (*n* = 2507). Furthermore, we excluded individuals who were born abroad with two Swedish-born parents (*n* = 1393), considering this group as ambiguous compared to our migrant and majority categories. We also excluded unaccompanied refugee minors (*n* = 967), because this group has been shown to differ in terms of psychiatric care utilisation, access to care and risk of mental health conditions compared to accompanied refugees and migrant youths (Axelsson *et al*., 2020). We also excluded individuals who had a psychiatric diagnosis prior to the study start (*n* =  28 781) and thus obtained a final cohort of 444 196 individuals.

### Participant consent

Consent is not required for register-based research under Swedish law. The identities of the study participants are unknown to the researchers.

### Exposure variables

Our exposure of interest was migration background. We defined migrants as individuals born abroad with at least one foreign-born parent (henceforth: migrants), descendants of migrants as Swedish-born individuals with at least one foreign-born parent (henceforth: descendants), and majority Swedish-born individuals as individuals born in Sweden with two Swedish-born parents (henceforth: Swedish). Among migrants, we defined refugees as those whose parents had received permanent residency as refugees by the Swedish Board of Migration, according to definitions from the United Nations refugee convention (stating that asylum as a refugee is granted to an individual who, ‘owing to a well-founded fear of being persecuted […] is unable to, or owing to such fear, is unwilling to avail himself of the protection of that country’) (UNHCR, 2011).

### Covariates

We considered age (categorised into three-year categories) and sex as potential confounders.

For parental income, we used annual disposable family income at the study start, categorised into quintiles in relation to the total population of each year, accounted for inflation. This variable is estimated by Statistics Sweden as an annual disposable income based on total family income (including welfare benefits), while weighting the total household income according to family composition and size, such that younger children are given lower weights than older household members and one adult is given a lower weight than two adults. For more information on this variable, please see the LISA-database documentation provided by Statistics Sweden (in Swedish only). When an individual had parents with differing income values, the rounded average was used.

### Secondary exposure

For migrants and descendants, we calculated parental time in Sweden based on parents' date of immigration and an individuals' date of entry into the study, derived from the parent with the longest time in Sweden. We categorised parental time in Sweden into four categories: 0–5 years, 6–10 years, 11–15 years and more than 15 years, using the latter as reference. When investigating the effect of parental time in Sweden, we excluded all Swedish individuals from our analyses, leaving 176 474 individuals in our analysis, and considered parental time as our exposure variable.

### Outcomes

We were interested in three outcomes: psychiatric diagnosis, level of care, and receipt of recommended treatments for specific diagnoses.

We studied *psychiatric diagnosis* given in any mental health service setting, including out-patient specialist psychiatric or paediatric care, in-patient psychiatric care and primary care. We used first-time diagnosis registered in any of the above settings in order to investigate differences in odds of receiving a specific psychiatric diagnosis between migrants, descendants and Swedish. All registers except the public child- and adolescent psychiatric clinic's register (BUP with Swedish acronym) use International Classification of Diseases, (ICD)-10 codes, whereas the BUP-register has its own system (based mainly on the Diagnostic and Statistical Manual of Mental Disorders, (DSM)-4 codes). BUP-diagnosis codes were categorised into equivalent ICD-10 codes (for a total list of how DMS-IV codes and the BUP-codes were translated into ICD-10, see online Supplementary material 1). The diagnoses studied and their ICD-10 codes were: Substance use disorder, F10-F19, F55.9, Psychotic disorder, F20-F29, F05, F06.0-2, Bipolar disorder, F30-F31, Mood disorder, F32-F34, F38-F39, F06.3, Mild/moderate depression, F320, F330, Severe depression, F322, F323, F332, F333, Anxiety disorder, F41, F400, F930, obsessive compulsive disorder (OCD) and body dysmorphic disorder (BDD), F42, F45.2A, post-traumatic stress disorder (PTSD), F431, F620, eating disorder, F50, sleep disorder, F51, intellectual disability, F70-F79, autism spectrum disorder, F84, attention deficit hyperactivity disorder (ADHD), F90, oppositional defiant disorder (ODD) and conduct disorder (CD), F913, F918, F919, Neurodevelopmental disorder, F70-F79, F84, F90, Self-harm and Injury with unclear intent, X60-X84, Y10-Y34.

*Level of care* was defined as the care setting where first-time diagnosis was set, i.e. whether the diagnosis was set in primary care, out-patient specialist psychiatric or paediatric care, or in-patient psychiatric care. We investigated potential differences between the exposure groups in terms of the type of care setting where first-time diagnosis was set, which we believe reflect access to specialist and primary care, as well as the severity of symptoms when reaching care.

*Receipt of recommended treatments for specific diagnoses*, mainly: psychotherapeutic and psychoeducational interventions, and psychotropic drug prescription. Here, we investigated whether migrant and descendant children had lower odds of receiving recommended treatments given a specific diagnosis, compared to Swedish children with the same diagnosis, using clinical guidelines to identify recommendations. When investigating recommended non-pharmaceutical treatments, we looked at diagnoses given exclusively within the out-patient child-and adolescent register (BUP), as it was from this register that we had data on such treatments.

### Treatment guideline sources

We identified three officially stated and regionally and nationally applied clinical guidelines, all in Swedish. For a list of these and a weblink to the guidelines, see online Supplementary material 2.

### Selection of diagnoses and associated treatments for investigation

From the BUP-register we had information regarding the treatment given to a patient, such as ‘family therapy’, ‘support in school’, ‘psycho-education’, etc. We categorised all psychotherapy-based treatments (e.g. cognitive behavioural therapy, psychodynamic therapy, interpersonal therapy, etc.) as an overall ‘psychotherapy’-category; family support interventions as a ‘family support’ category; family therapy treatments as a ‘family therapy’-category, and psychoeducational interventions as a distinct category. We also investigated parental training, group therapy interventions, and social training interventions, when these were stipulated as recommended interventions.

We decided to investigate diagnoses with precise guidelines where the first recommended steps were either psychotherapeutic, psycho-educational/psycho-supportive or pharmaceutical interventions. These diagnoses included: anxiety disorder, depression, obsessive-compulsive disorder and dysmorphophobia, PTSD, ODD and CD, ADHD, psychotic disorder, and sleep disorder with serious comorbidity.

For each diagnosis, we first identified all treatments given within three months of diagnosis. We then identified the recommended treatments for each specific diagnosis and dichotomised the outcome as having received or not received the recommended treatment, within three months of diagnosis, for the diagnosis under scrutiny. We included first-step recommendations, such as psychoeducation and second-step recommendations, such as psychotherapy, as separate outcomes, and investigated whether any of these had been given three months within diagnosis.

For *anxiety disorder* and *mild to moderate depression*, guidelines stipulate a first intervention consisting of parental support and psycho-pedagogical interventions, such as information about the condition and strategies for creating stability in the home and school environment, followed by psychotherapeutic interventions as the next step.

For *ODD/CD*, parental support and individual or family psychotherapeutic interventions, as well as social skills training, are primary recommendations.

For *OCD/BDD and PTSD*, the primary recommendation is cognitive behavioural therapy.

We investigated psychoeducational and parental support interventions given an anxiety disorder diagnosis or a diagnosis of mild/moderate depression (reported as ‘support, anxiety disorder’ and ‘support, mild/mod depression’, respectively, in Fig. 3); individual or group therapy given an anxiety disorder diagnosis or a diagnosis of mild/moderate depression (reported as ‘psychotherapy anxiety disorder’ and ‘psychotherapy mild/mod depression’, respectively, in Fig. 3); family support, individual or family psychotherapy interventions, and social skills training given a diagnosis of ODD/CD (reported as ‘treatment, ODD/CD’, in Fig. 3); and psychotherapy given a diagnosis of PTSD or OCD/BDD (reported as ‘psychotherapy PTSD’ and ‘psychotherapy, OCD/BDD’, respectively, in Fig. 3).

For diagnoses where pharmaceutical treatment was recommended, we dichotomised the outcome as has having received or not received a prescription of the medication recommended for this diagnosis. We investigated anti-depressive medication (ATC-code N06B) for severe depression, ADHD-medication (ATC-code: N06B) for ADHD, and neuroleptics (ATC-code N05A) for psychotic disorder. We also investigated psychotropic drug treatment of sleep disorder with serious comorbidity (bipolar or psychotic disorder), as guidelines here recommend pharmacological treatment. Here, we looked at sedatives (ATC-code: N05C and R06AD; the latter group covering antihistamines recommended as sedatives in the pharmacological treatment guidelines). For anxiety disorder, we looked at anti-depressants, sedatives (including antihistamines) and tranquilisers, given that the instructions propose primarily SSRI-medication, and if clinically warranted, certain sedatives and tranquilisers (e.g. prometazin and hydroxizin) for treatment of severe anxiety disorder. Please see online Supplementary material 7 for a table of recommended treatments for the investigated disorders.

### Statistical methods

We report basic demographic characteristics, using chi-square tests to compare demographic variables and diagnoses in our study population (see Table 1). We used logistic regression models to estimate odds ratios (ORs) and 95% confidence intervals (CIs) for our different outcomes, adjusting for age in all analyses, and for sex and parental income in our full model. We used SAS 9.4 for data management and Stata MP16 for descriptive statistics and regression analyses.

**Table 1.** Characteristics of the study population and psychiatric diagnosis at first visit, percentages

Study population					
*N* = 444 196	Swedish %	Descendents %	Migrants %	Refugees %	*p*-Value
	60.3	31.4	7.1	1.3	
Parental income quintile					
Lowest	3.2	24.7	51.8	74.8	<0.001
Medium lowest	13.5	29.4	25.2	18.2	
Medium	28.5	23.9	11.2	5.3	
Medium highest	34.0	15.4	6.9	1.3	
Highest	20.7	6.5	5.0	0.4	
Age group (years of age)					
6 to 8	65.0	68.1	44.8	35.9	<0.001
9 to 11	13.7	13.2	20.9	20.8	
12 to 14	10.5	9.5	14.1	16.8	
15 to 17	10.8	9.2	20.2		
Sex					
Males	51.0	50.7	50.4	51.2	0.094
Females	49.0	49.3	49.6	48.8	
Parental time in Sweden					
0–5 years	N/A	5.2	72.1	71.9	<0.001
5–10 years	N/A	7.0	12.4	18.0	
10–15 years	N/A	12.6	5.1	7.4	
15 or more years	N/A	75.2	10.4	2.8	
Diagnosis at first visit					
Substance use disorder	1.2	1.1	1.0	0.9	0.046
Psychotic disorder	<1	0.1	0.1	0.2	<0.001
Bipolar disorder	0.1	0.1	0.1	<1	0.34
Mood disorder	2.4	1.7	1.4	1.3	<0.001
Anxiety disorder	3.1	2.3	1.8	1.9	<0.001
Post traumatic stress disorder	0.2	0.3	0.5	1.1	<0.001
Obessessive compulsive disorder, body dysmorphic disorder	0.6	0.4	0.2	0.2	<0.001
Anxiety disorder	3.1	2.3	1.7	1.9	<0.001
Eating disorder	0.7	0.5	0.3	0.4	<0.001
Sleep disorder	0.5	0.4	0.4	0.5	<0.001
Neurodevelop-mental disorder	5.5	5.3	3.6	3.5	<0.001
Oppositional defiant disorder, conduct disorder	0.8	0.9	0.6	0.8	<0.001
Selfharm	1.0	0.9	0.8	1.0	0.017
Unspecified	0.4	0.3	0.3	0.2	0.061
Comorbidity	33.2	30.1	27.8	25.7	<0.001

## Results

The study population consisted of 444 196 individuals. 267 650 (60.3%) were Swedish, 139 402 (31.4%) were descendants, 31 405 (7.1%) were non-refugee migrants and 5739 (1.3%) were refugees. There were significant differences between the groups in terms of age, parental income and parental time in Sweden, but not in terms of sex (see Table 1).

**Fig. 1.**
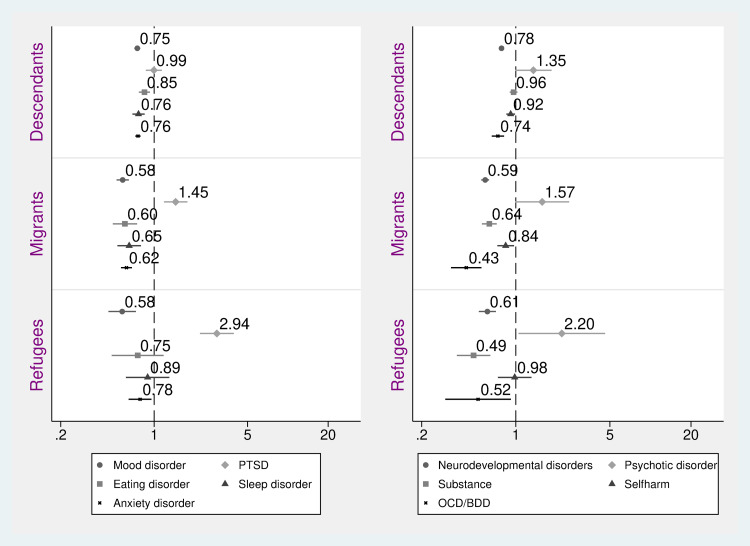
Odds ratios and 95% confidence intervals of first-time psychiatric diagnosis among children and adolescents, comparing migrant subgroups and descendants with Swedish (reference). Adjusted for age, sex and parental income.

Figure 1 shows ORs and 95% CIs for first-time diagnosis of mood disorder, anxiety disorder, PTSD, sleep disorder, eating disorder, neurodevelopmental disorder (ADHD, autism-spectrum disorder and intellectual disability), self-harm (including injury with unclear intent), psychotic disorder, substance use disorder, OCD/BDD, and ODD/CD, in any care setting (i.e. out-patient psychiatric or paediatric care, primary care or in-patient psychiatric care).

Results from the fully adjusted models show that descendants had a slightly, yet statistically significant, lower likelihood to be diagnosed with a mood disorder, anxiety disorder, eating and sleep disorders, OCD/BDD and neurodevelopmental disorder, compared to Swedish (Fig. 1). For diagnosis with PTSD, self-harm, psychotic disorder and substance use disorder we observed no significant differences between descendants and Swedish, and the point estimates were, except for psychotic disorder, close to the reference (Fig. 1).

Non-refugee and refugee migrants alike had significantly lower ORs of being diagnosed with nearly all the investigated diagnoses, including anxiety disorder, mood disorder, neurodevelopmental disorders, substance use disorder and OCD/BDD (see Fig. 1). For eating disorder, sleep disorder, self-harm and bipolar disorder, results showed lower OR of receiving these diagnoses for non-refugee migrants, compared to Swedish, but no significant difference between refugees and the reference group (Fig. 1; data on the latter diagnosis not shown). In contrast, both non-refugee and refugee migrants had higher ORs of receiving a diagnosis of PTSD, and refugee migrants had higher ORs of receiving a psychotic disorder diagnosis. Adjusting for sex and parental income altered results such that the OR of PTSD for descendants changed from significant estimates to non-significant estimates, while differences overall were attenuated but remained significant (see online Supplementary material 3 for adjusted for age-only-results).

**Fig. 2.**
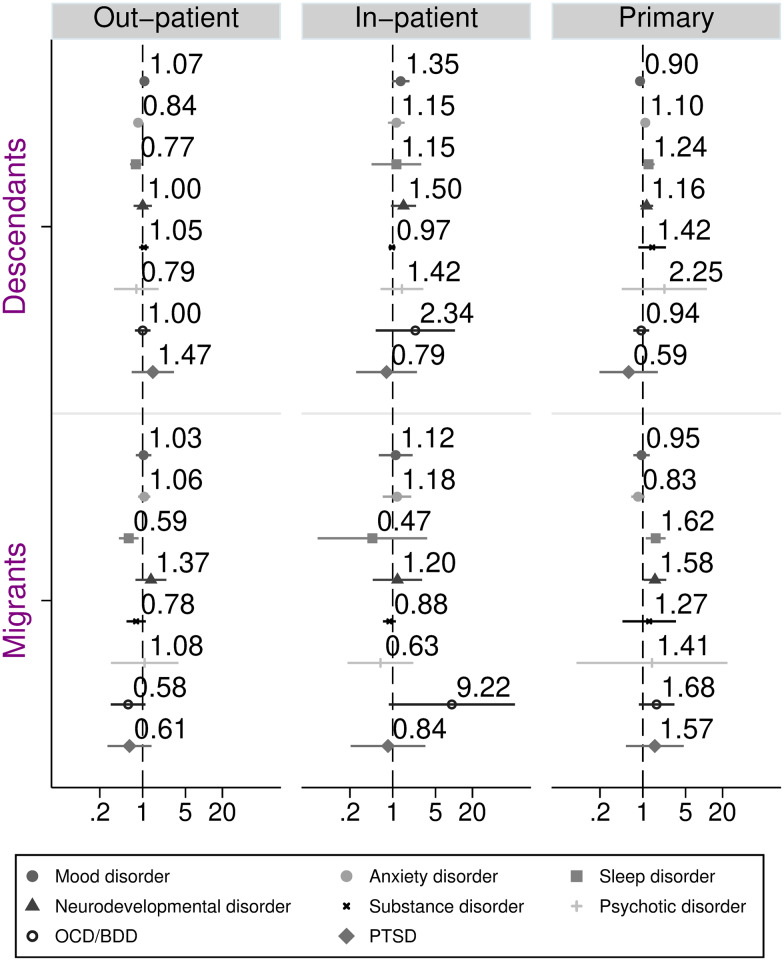
Odds ratios and 95% confidence intervals of first-time diagnosis in different care settings among children and adolescents, comparing migrants and descendants with Swedish (reference). Adjusted for age, sex and parental income.

Next, we investigated ORs of receiving, for the first time, a specific diagnosis in different care settings (i.e. out-patient specialist care, in-patient specialist care or primary care) for migrants (including refugees) and descendants, as compared to Swedish, see Fig. 2. Results showed that there were no significant differences between migrants and descendants, as compared to Swedish, in terms of the level of care where a first-time diagnosis was set for a mood disorder, substance use disorder, PTSD, OCD/BDD and psychotic disorder. However, for a sleep disorder, results showed a slightly decreased likelihood of diagnosis in out-patient care and increased risk of diagnoses in primary care among migrants and descendants, compared to Swedish, see Fig. 2. (Eating disorder and self-harm not shown; non-significant results.) Shifting from partial to full adjustment did not alter the results (partially adjusted data not shown).

**Fig. 3.**
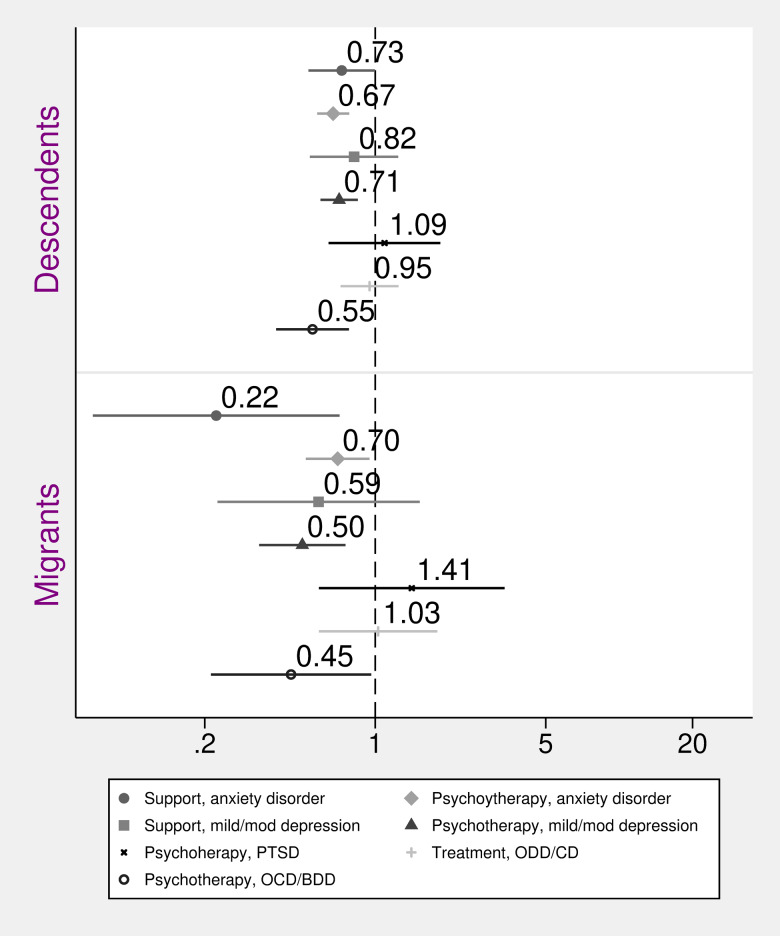
Odds ratios and 95% confidence intervals of recommended treatments for selected diagnoses among children and adolescents, comparing migrants and descendants with Swedish (reference). Recommended treatments included psychoeducational and parental support interventions for anxiety disorder or mild/moderate depression (‘support, anxiety disorder’ and ‘support, mild/mod depression’); individual or group therapy for anxiety disorder or mild/moderate depression (‘psychotherapy, anxiety disorder’ and ‘psychotherapy, mild/mod depression’); family support, individual or family psychotherapy, and social skills training for ODD/CD (‘treatment, ODD/CD’); psychotherapy for PTSD or OCD/BDD (‘psychotherapy, PTSD’ and ‘psychotherapy, OCD/BDD’). Adjusted for age, sex and parental income.

Figure 3 shows the ORs of receiving recommended treatments for anxiety disorder, mild to moderate depression, PTSD, ODD/CD and OCD/BDD, for descendants and migrants. These analyses were based on diagnoses from the BUP-register. There were 29 486 individuals with a registered first visit to BUP, and among these, 13 065 had received any of the above-mentioned diagnoses whose recommended treatments were investigated. Results show that the OR of receiving recommended treatments were lower for both groups, compared to Swedish, for various examined treatment outcomes. The OR of receiving psychotherapy for anxiety disorder, for mild/moderate depression, and for OCD/BDD was significantly lower for descendants and migrants, respectively (see Fig. 3), and the pattern was the same for receiving parental support/psycho-education for anxiety disorder among migrants. Adjusting for sex and parental income attenuated these results, but they remained statistically significant (see online Supplementary material 4 for adjusted for age-only-results).

**Fig. 4.**
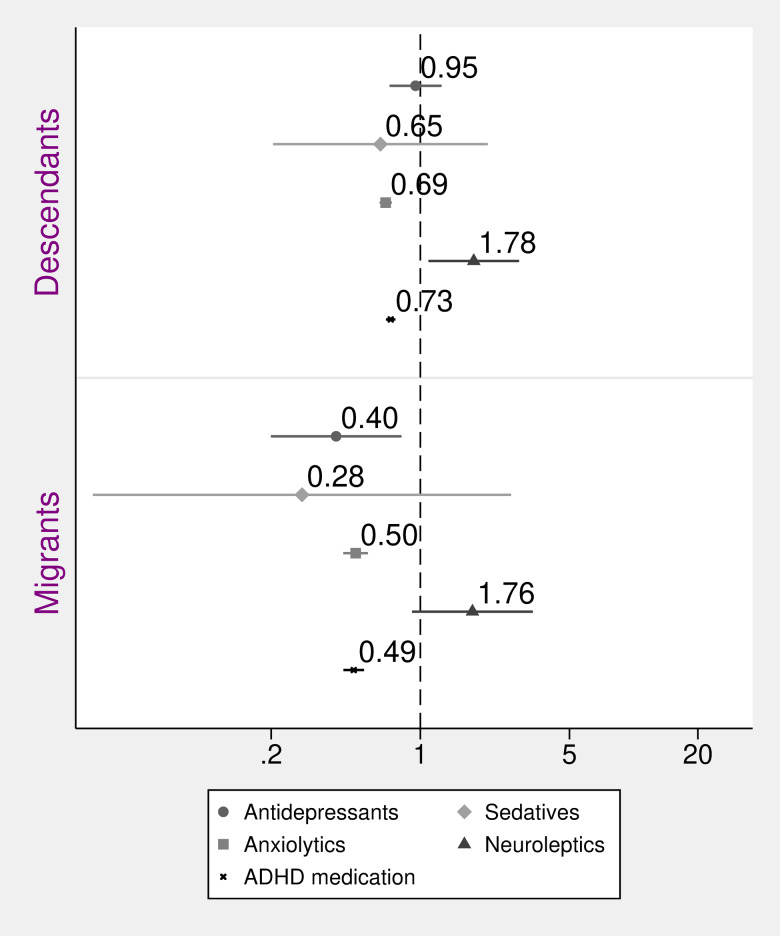
Odds ratios and 95% confidence intervals of recommended pharmaceutical treatments for selected diagnoses among children and adolescents, comparing migrants and descendants with Swedish (reference). Adjusted for age, sex and parental income.

Figure 4 shows the ORs of receiving recommended pharmacological treatments for ADHD, sleep disorder with comorbidity, psychotic disorder, severe depression and anxiety disorder. Results show that, for both migrants and descendants, the OR of receiving ADHD-medication given an ADHD-diagnosis, and for receiving anxiolytics given an anxiety disorder diagnosis, were significantly lower compared to Swedish (see Fig. 4), while the OR for receiving neuroleptics given a psychotic disorder diagnosis was higher among descendants. There were no significant differences between migrants and Swedish in terms of odds of receiving neuroleptics given a diagnosis of psychosis, nor for receiving medication for a sleep disorder with serious comorbidity, see Fig. 4. Adjusting for sex and parental income altered only the results concerning neuroleptics, such that the OR was attenuated post adjustment (see online Supplementary material 5 for adjusted for age-only-results).

Lastly, we investigated whether parental time in Sweden affected the likelihood of receiving specific diagnoses and recommended treatments, for outcomes where we had observed differences between our exposure groups. We observed that the OR of receiving a diagnosis of ADHD, anxiety disorder and mood disorder, as well as the OR of receiving ADHD-medication given a diagnosis of ADHD and anxiolytics given a diagnosis of anxiety disorder was lower for all groups with less than 15 years of parental time in Sweden. However, the OR of receiving psychotherapy for anxiety disorder and for OCD/BDD did not differ significantly with parental time in Sweden. Furthermore, the OR of receiving antidepressants for severe depression and psychotherapy treatment for mild/moderate depression was lower for those whose parents had been in Sweden for 0–5 years, while those with a parental time of 10–15 years did not differ significantly from the reference group. In contrast, those whose parents had been the shortest time in Sweden had higher odds of receiving a PTSD diagnosis, compared to those whose parents had been in Sweden for more than 15 years, see online Supplementary material 6.

Given the heterogeneity among migrant and descendent children with differing parental time in Sweden, as well as the fact that parental region of origin has been shown to affect utilisation (Ivert *et al*., 2013), we conducted two additional analyses to address this point. First, we adjusted for a maternal region of origin, categorised into ten world regions by Statistics Sweden, using the Nordic countries as reference (Sweden, Denmark, Finland, Iceland and Norway), and found that estimates did not significantly change with this adjustment (see online Supplementary material 8). Second, we investigated the odds of treatment and diagnosis receipt among migrants and descendants, using maternal region of origin as the main exposure and adjusting for a parental time in Sweden, parental income, age and sex (see online Supplementary material 9). Here, we observed that children with mothers born in Africa South of Sahara, the Middle East and North Africa had lower odds of receiving all investigated treatments and diagnoses compared to children with Swedish-born mothers, except for receipt of therapy for OCD/BDD and PTSD-diagnosis, for which no significant differences were observed. Children with mothers born in Asia had significantly lower odds of receiving a diagnosis of ADHD, mood disorder and anxiety disorder, as well as the recommended treatments for these disorders, while children with mothers from South America had higher odds for receiving a PTSD-diagnosis and mood disorder diagnosis, see online Supplementary material 9.

## Discussion

We studied differences between migrants and descendants, as compared to Swedish majority children, in terms of receiving a specific psychiatric diagnosis, level of care where a diagnosis was set, and of receiving recommended treatments given a specific diagnosis. In line with our hypotheses, our findings demonstrate that migrant and descendent children are less likely to be diagnosed with several psychiatric conditions; that when in contact with mental health services, they are less likely to receive recommended treatments for certain conditions; and, that parental time and reason for migration explain some of the differences we expected to find. However, the hypothesis that there would be differences in where the groups were diagnosed was rejected.

Both non-refugee and refugee migrant children had a lower likelihood of being diagnosed with mood disorder, anxiety disorder and neurodevelopmental conditions, indicating an underutilisation of services in line with previous research (Ivert *et al*., 2013; Barghadouch *et al*., 2016; Berg *et al*., 2020). It is especially concerning to observe the lower likelihood of a diagnosis of neurodevelopmental conditions, given that research suggests that these conditions may be under-diagnosed (Morinaga *et al*., 2020). For adults, research has shown mismatches between culturally affected expressions of distress and conventional psychiatric diagnostic tools among ethnic and cultural minorities (Bhui *et al*., 2004; Lewis-Fernández *et al*., 2010). For children, more research is needed to investigate these issues. A previous case-note study comparing refugee children to Norwegian children showed that refugee children were diagnosed more frequently with PTSD and other affective and emotional disorders, and less often with pervasive developmental disorders and ADHD (Vaage *et al*., 2007). According to the authors, refugee children may more often be understood as traumatised, while neurobiological disorders may be overlooked (Vaage *et al*., 2007). Perhaps a similar influence of migration background is at play in our findings. However, other studies point to a good validity of adult PTSD in the registers used (Hollander *et al*., 2019), and hence, more research is needed to test if, and how, migration background influences diagnostic practices for children.

We observed few differences between migrants and descendants, as compared to Swedish, in terms of the level of care where a first psychiatric diagnosis was set. Previous research has indicated that migrant youth utilise more in-patient and emergency psychiatric services, and less out-patient psychiatric care, compared to the majority individuals (Barghadouch *et al*., 2016; Manhica *et al*., 2017; de Montgomery *et al*., 2020). Contrary to our hypothesis, we observed no such differences. This could imply that, for those migrant and descendent children who do reach mental health services, barriers to out-patient care may not be as predominant as we had hypothesised. However, it may also be the case that since we measured diagnoses at first contact, we failed to detect differences that would have emerged had we also investigated the total number of visits.

To the best of our knowledge, this is the first study to investigate in detail the receipt of recommended treatments among migrant and descendent children. Our findings demonstrate that migrant and descendant children had a lower likelihood of receiving several recommended treatments, compared to Swedish majority children with the same diagnosis. Thus, we found that migrant and descendant children had a lower likelihood of receiving psychotherapeutic treatments for anxiety disorder, mild/moderate depression and OCD/BDD, compared to their Swedish peers, though clinical guidelines specify these as the recommended interventions. Moreover, we observed a lower likelihood for receiving ADHD-medication given an ADHD-diagnosis, anti-depressants given a diagnosis of severe depression, and anxiolytics given a diagnosis of anxiety disorder, among migrant and descendant children compared to the reference group.

Consequently, migrants and descendants with a diagnosis of severe depression, anxiety disorder and ADHD appear to be at risk of not receiving clinically recommended treatments. Parental time in Sweden did not unequivocally explain the differences observed. Our findings suggest that the likelihood of receiving an ADHD-diagnosis, ADHD-treatment, anxiety disorder diagnosis, anxiolytics and a mood diagnosis, was lower for those with less than 15 years of parental residency, partly in line with studies showing increased use of psychiatric care with increased residency (Brendler-Lindqvist *et al*., 2014), while parental residency time did not appear to affect the receipt of psychotherapy for anxiety disorder and depression. Similarly, parental income did not explain our findings of disparities in receipt of recommended treatments to any substantial extent.

The discrepancies in terms of treatment receipt were larger between migrant and Swedish majority children, than between descendent and Swedish majority children, which is consistent with findings that psychiatric care use is higher among descendent refugee children compared to newly arrived refugee children (Berg *et al*., 2020). Given that research has shown that migrant children may face multiple barriers to psychiatric care, including communication and cultural barriers, as well as limited knowledge of the mental health care system among parents (Place *et al*., 2021), it is conceivable that families with children who are born in Sweden may be better able to navigate the mental health care system and to overcome barriers, compared to more recently arrived migrant families. Lack of knowledge of the Swedish language should not formally restrict the receipt of certain types of treatments, such as psychotherapy, which should be offered with the help of interpreters, but such obstacles may of course nonetheless exist.

Moreover, we found that maternal region of origin influenced the likelihood of receiving several diagnoses and treatments, also when adjusting for a parental time in Sweden. Children with mothers from Sub-Saharan Africa, the Middle East and North Africa appear to be at the highest risk for underutilisation and sub-optimal treatment receipt, compared to children with Swedish-born mothers. These findings are partly in line with previous research indicating that adolescents with parents from low-income countries, where child psychiatric services are scarce (Ivert *et al*., 2013; Berg *et al*., 2020), may have difficulties accessing mental health care in Sweden. Our findings suggest that such barriers may also affect the provision and receipt of recommended psychiatric treatments.

Previous studies suggest that attitudes and perceptions among parents affect the use of psychiatric services among migrant youths (Verhulp *et al*., 2015). Immigrant parents may be less likely to accept psychotropic drug treatments compared to the majority parents (Guzder *et al*., 2013), while psychotherapy adapted to transcultural encounters may better meet the needs of migrant children (Grau *et al*., 2020). Most studies, however, have focused on barriers to accessing care, and therefore, we do not know how saliently reported barriers such as stigma, language obstacles, lack of knowledge of services, perceived lack of cultural sensitivity among providers, etc., (Place *et al*., 2021) relate to the provision and/or acceptance of treatments for those who overcome barriers to accessing care.

### Limitations and future directions

The study includes the entire population of children aged 6–17 years living in the Stockholm Region between 2006 and 2015. The use of register-based data allows for a comprehensive examination of psychiatric care. These two factors make these findings generalisable to its population with strong validity. Some limitations should be noted, however. First, using register data, we do not have information on the self-reported need, making our interpretations regarding potential under-utilisation and under-diagnosis tentative. Also, we had no information on prior diagnoses of newly immigrated individuals, whose previous contact with care could thus not be taken into account. This may be considered a potential bias, as newly arrived immigrants with prior mental health disorders, who may have received care in their home countries, could not be excluded. Such a potential prior contact with care could contribute to a stronger familiarisation with services, and as such, possibly facilitate help-seeking once in Sweden. We considered the potential bias from prior diagnoses received in Sweden and within the Swedish mental health care system, to be of greater importance, however, and therefore opted for the chosen approach, while recognising its potential shortcomings.

We tried to capture inequalities in treatments received for the same diagnosis across our exposure groups, but conclusions should be drawn cautiously. It is crucial to bear in mind that we have information only on treatments that have been provided and lack data on treatments that have been offered but rejected. Moreover, though we refer to clinical guidelines, there could be clinically sound reasons for not offering an individual patient recommended treatments, even when guidelines stipulate otherwise. Lastly, investigating not only the first diagnosis, but the total number of visits, diagnoses and treatments would provide a more comprehensive picture of utilisation patterns, as an investigation into comorbidities and their treatments would allow for a more complete analysis of discrepancies in service use and whether the observations we have made remain or change when taking later diagnoses into full account.

Notwithstanding these limitations, we believe that our study adds valuable knowledge about discrepancies in mental health care utilisation, and notably, in the receipt of psychiatric treatments, among migrant and descendent children, in a research area that has so far been little investigated. More research is needed in order to better understand disparities in treatment provision for mental health disorders, and the reasons behind these discrepancies. In addition, more knowledge – and action – is required in order to develop policies that ensure equal access to, and quality of, mental health care for all children and youth in Sweden. Future research should focus on cross-cultural validation studies of psychiatric diagnoses and symptom variations, as well as on potential cultural and linguistic barriers that may impede the equitable provision and receipt of recommended treatments.

## Data Availability

The data used in this study cannot be made publicly available according to Swedish data protection law. Any questions about the data can be addressed to the corresponding author. The statistical code is available from the corresponding author.

## Supplementary material

For supplementary material accompanying this paper visit http://doi.org/10.1017/S2045796022000142. click here to view supplementary material
