# Supplementary material for: Are all children treated equally? Psychiatric care and treatment receipt among migrant, descendant and majority Swedish children: a register-based study
Source: Epidemiol Psychiatr Sci. 2022 Apr 19;31:e20. doi: 10.1017/S2045796022000142 (PMC9069577; doi:10.1017/S2045796022000142)
Supplement: Supplementary file 1 [file S2045796022000142sup001.zip › Supplementary material 9_for_revision.docx]

**Supplementary material 9. Odds ratios of specific diagnoses and specific recommended treatments among migrant and descendant children with different maternal regions of origin (reference: Swedish origin), adjusted for parental time in Sweden, parental income, age and sex**

*OCD/BDD/tics=obsessive compulsive disorder/body dysmorphic disorder

|  | ADHD diagnosis | ADHD medication (given a diagnosis of ADHD) | Anxiety disorder diagnosis | Therapy treatment (given diagnosis of anxiety syndrome) | Anxiolytics (given diagnosis of anxiety syndrome) | Mood disorder diagnosis | Therapy treatment (given diagnosis of mild to moderate depression) | Antidepressants (given diagnosis of severe depression) | Therapy treatment given (diagnosis of OCD/BDD*) | PTSD diagnosis |
| --- | --- | --- | --- | --- | --- | --- | --- | --- | --- | --- |
| Mother’s region of origin | OR and 95% CI | OR and 95% CI | OR and 95% CI | OR and 95% CI | OR and 95% CI | OR and 95% CI | OR and 95% CI | OR and 95% CI | OR and 95% CI | OR and 95% CI |
| Africa south of Sahara | **0.59 (0.54-0.65)** | **0.50 (0.43- 0.58)** | **0.29**  **(0.24-0.34)** | **0.28 (0.15-0.53)** | **0.22 (0.17-0.29)** | **0.26 (0.21-0.32)** | **0.22 (0.09- 0.50)** | **0.17 (0.04-0.73)** | 0.22 (0 .04-0.94) | **0.64 (0.45-0.89)** |
| Asia | **0.43 (0.39- 0.48)** | **0.45 (0.39-0.53)** | **0.50**  **(0.44-0.57)** | **0.57 (0.37-0.87)** | **0.42 (0.35-0.52)** | **0.62 (0.54-0.71)** | 0.79 (0.50- 1.23) | 0.78 (0.38-1.57) | **0.19 (0.05-0.81)** | 0.91 (0.67-1.22) |
| Eastern EU, RUS&Baltics | **0.58 (0.53-0.64)** | **0.58 (0.50- 0.67)** | **0.68**  **(0.61-0.77)** | 0.85 (0.59-1.24) | **0.63 (0.52- 0.75)** | **0.65 (0.57- 0.75)** | 0.64 (0.39- 1.05) | 0.88 (0.44-1.77) | 0.62 (0.25-1.56) | **0.65 (0.47-0.91)** |
| Middle East and North Africa | **0.63 (0.59- 0.68)** | **0.63 (0.57-0.70)** | **0.61 (0.56- 0.67)** | **0.58 (0.40-0.82)** | **0.49 (0.42-0.56)** | **0.49 (0.43-0.55)** | **0.25 (0.15- 0.44)** | **0.36 (0.17-0.73)** | 0.47 (0.22 1.01) | 0.88 (0.67-1.13) |
| South America | 1.06 (0.97- 1.17) | 1.02 (0.89-1.16) | 1.06 (0.94-1.18) | 1.35 (0.93-1.94) | 0.92 (0.77-1.09) | **1.16 (1.02-1.31)** | 1.31 (0.86- 2.00) | 1.36 (0.71-2.59) | 0.76 (0.29-1.97) | **1.47 (1.08-2.00)** |
| USA, Canada and Oceanic | 0.90 (0.72-1.13) | 0.98 (0.72- 1.33) | 0.87 (0.66-1.14) | 0.34 (0.08-1.40) | 0.65 (0.42-1.02) | **0.66 (0.47-0.93)** | 0.99 (0.36- 2.71) | 0.62 (0.08-4.57) | No cases | 0.29 (0.07-1.19) |
| Western&Southern Europe | **0.69 (0.60- 0.80)** | **0.64 (0.52 0.79)** | **0.68 (0.57 0.80)** | **0.52 (0.27-0.99)** | **0.65 (0.50-0.80)** | **0.67 (0.55-0.81)** | 0.75 (0.39- 1.46) | 0.57 (0.17-1.84) | 0.25 (0.03- 1.86) | **0.54 (0.31-0.94)** |
