# Supplementary material for: Are all children treated equally? Psychiatric care and treatment receipt among migrant, descendant and majority Swedish children: a register-based study
Source: Epidemiol Psychiatr Sci. 2022 Apr 19;31:e20. doi: 10.1017/S2045796022000142 (PMC9069577; doi:10.1017/S2045796022000142)
Supplement: Supplementary file 1 [file S2045796022000142sup001.zip › Supplementary material 8_for_revision.docx]

**Supplementary material 8. Odds ratios of specific diagnoses and specific recommended treatments among migrant and descendant children with different parental time of residence in Sweden (reference: more than 15 years of parental residence in Sweden). Adjusted for age, sex, parental income and maternal region of origin.**

*OCD/BDD/tics=obsessive compulsive disorder/body dysmorphic disorder

|  | ADHD diagnosis | ADHD medication (given a diagnosis of ADHD) | Anxiety disorder diagnosis | Therapy treatment (given diagnosis of anxiety syndrome) | Anxiolytics (given diagnosis of anxiety syndrome) | Mood disorder diagnosis | Therapy treatment (given diagnosis of mild to moderate depression) | Antide-pressants (given diagnosis of severe depression) | Therapy treatment given (diagnosis of OCD/BDD*) | PTSD diagnosis |
| --- | --- | --- | --- | --- | --- | --- | --- | --- | --- | --- |
| Parental time i Sweden | OR and 95% CI | OR and 95% CI | OR and 95% CI | OR and 95% CI | OR and 95% CI | OR and 95% CI | OR and 95% CI | OR and 95% CI | OR and 95% CI | OR and 95% CI |
| 0-5 years | **0.70 (0.65-0.76)** | **0.62 (0.55-0.70)** | **0.75**  **(0.68-0.84)** | 0.93 (0.65-1.33) | **0.66 (0.56-0.79)** | **0.75 (0.66- 0.84)** | **0.59 (0.35- 0.99)** | **0.37 (0.15-0.88)** | 0.51 (0 .19-1.38) | **1.93 (1.57-2.37)** |
| 6-10 years | **0.67 (0.60- 0.75)** | **0.63 (0.53-0.74)** | **0.65**  **(0.56-0.76)** | 0.77 (0.47-1.28) | **0.63 (0.49-0.80)** | **0.82 (0.72-0.96)** | 0.65 (0.33- 1.28) | 0.59 (0.21-1.65) | 0.86 (0.15-2.47) | 0.74 (0.51-1.08) |
| 11-15 years | **0.83 (0.75-0.90)** | **0.77 (0.67- 0.88)** | **0.82**  **(0.72-0.93)** | 0.94 (0.62-1.42) | 0.91 (0.76- 1.09) | **0.70 (0.59- 0.83)** | 0.99 (0.59- 1.64) | 0.76 (0.33-1.70) | 0.50 (0.15-1.67) | 0.96 (0.72-1.31) |
