# Supplementary material for: Are all children treated equally? Psychiatric care and treatment receipt among migrant, descendant and majority Swedish children: a register-based study
Source: Epidemiol Psychiatr Sci. 2022 Apr 19;31:e20. doi: 10.1017/S2045796022000142 (PMC9069577; doi:10.1017/S2045796022000142)
Supplement: Supplementary file 1 [file S2045796022000142sup001.zip › Supplementary material 7_for_revision.docx]

**Supplementary material 7. Table of recommended treatments for specific disorders**

| **Diagnosis** | **Recommended non-pharmacological treatment step 1** | **Recommended non-pharmacological treatment step 2** | **Pharmacological treatment as complementary or primary recommendation** |
| --- | --- | --- | --- |
| Psychotic disorder, F20-F29, F05, F06.0-2 |  |  | Neuroleptics (ATC-code N05A) |
| Mild or moderate depression  F320, F330 | Parental support  Psycho-pedagogical interventions (e.g. psychoeducation, problem solving communication training) | Psychotherapy (individual or group cognitive behavioral therapy) |  |
| Severe depression, F322, F323, F332, F333 | Anti-depressive medication (ATC-code N06B) |  |  |
| Anxiety disorder  (F41, F400, F930) | Psychoeducation  Parental support | Psychotherapy  (cognitive behavioral therapy) | Anxiolytics (ATC-code N05B) |
| Obsessive-compulsive disorder & dysmorphophobia  F42, F45.2A | Cognitive behavioral therapy |  |  |
| Post traumatic stress disorder  F431, F620 | Cognitive behavioral therapy |  |  |
| Oppositional defiant disorder and conduct disorder  F913, F918, F919, | Family support  Social skills training  Individual or family psychotherapy (cognitive behavioral therapy) |  |  |
| Attention deficit and hyperactivity disorder (F90) |  |  | ADHD-medication (ATC-code: N06B) |
| Sleep disorder with serious comorbidity |  |  | Sedatives (ATC-code: N05C and R06AD) |
