# Supplementary material for: Are all children treated equally? Psychiatric care and treatment receipt among migrant, descendant and majority Swedish children: a register-based study
Source: Epidemiol Psychiatr Sci. 2022 Apr 19;31:e20. doi: 10.1017/S2045796022000142 (PMC9069577; doi:10.1017/S2045796022000142)
Supplement: Supplementary file 1 [file S2045796022000142sup001.zip › supplementary material 6 (parental time).pdf]

	ADHD diagnosis	ADHD medication (given diagnosis of ADHD)	Anxiety disorder diagnosis	Therapy treatment (given diagnosis of anxiety syndrome)	Anxiolytics (given diagnosis of anxiety syndrome)	Mood disorder diagnosis	Therapy treatment (given diagnosis of mild to moderate depression)	Anti-depressants (given diagnosis of severe depression)	Therapy treatment given (diagnosis of OCD/BDD*)	PTSD diagnosis
Parental time in Sweden	OR and 95% CI	OR and 95% CI	OR and 95% CI	OR and 95% CI	OR and 95% CI	OR and 95% CI	OR and 95% CI	OR and 95% CI	OR and 95% CI	OR and 95% CI
0-5 years	**0.60 (0.55-0.65)**	**0.53 (0.47-0.60)**	**0.66 (0.60-0.73)**	0.82 (0.59-1.15)	**0.58 (0.49-0.67**	**0.65 (0.58-0.73)**	**0.57 (0.35-0.92)**	**0.36 (0.15-0.82)**	0.40 (0.15-1.04)	**1.76 (1.46-2.14)**
6-10 years	**0.58 (0.52-0.65)**	**0.58 (0.52-0.64)**	**0.57 (0.49-0.66)**	0.70 (0.43-1.14)	**0.52 (0.41-0.66)**	**0.60 (0.50-0.71)**	0.53 (0.27-1.04)	0.50 (0.18-1.38)	0.65 (0.23-1.85)	0.74 (0.52-1.05)
11-15 years	**0.69 (0.63-0.75)**	**0.69 (0.63-0.75)**	**0.65 (0.58-0.73)**	0.75 (0.50-1.11)	**0.69 (0.41-0.66)**	**0.63 (0.55-0.73)**	0.70 (0.43-1.14)	0.58 (0.26-1.26)	0.36 (0.11-1.16)	0.86 (0.65-1.15)

Supplementary material 6. Odds ratios of specific diagnoses and specific recommended treatments among migrant and descendant children with different parental time of residence in Sweden (reference: more than 15 years of parental residence in Sweden)

*OCD/BDD/tics=obsessive compulsive disorder/body dysmorphic disorder
