# Supplementary material for: Are all children treated equally? Psychiatric care and treatment receipt among migrant, descendant and majority Swedish children: a register-based study
Source: Epidemiol Psychiatr Sci. 2022 Apr 19;31:e20. doi: 10.1017/S2045796022000142 (PMC9069577; doi:10.1017/S2045796022000142)
Supplement: Supplementary file 1 [file S2045796022000142sup001.zip › Supplementary material 2 (clinical guidelines).pdf]

Supplementary material 2

Clinical guidelines

i) Child and adolescent psychiatry Stockholm (in Swedish, BUP Stockholm) book of guidelines, called "***Guidelines to support evaluation and treatment 2015***" (in Swedish "Riktlinjer till stöd för bedömning och behandling").
This book of guidelines builds on earlier versions published in 2010, 2012 and 2015, and was developed by clinicians within the child and adolescent psychiatric clinics in the Stockholm Region (BUP Stockholm).

The book can be accessed and downloaded from here:
https://registercentrum.blob.core.windows.net/qbup/r/Riktlinjer-till-st-d-f-r-bed-mning-och-behandling-2015-Bks8u9ciZ.pdf

ii) Child and adolescent psychiatry Stockholm (BUP Stockholm) "***Instructions for child and adolescent psychiatric pharmacological treatments***" (in Swedish, Instruktion för barn- och ungdomspsykiatrisk läkemedelsbehandling"), developed by child and adolescent psychiatrists, aimed to provide clinicians with guidelines for pharmacological treatment of specific diagnoses, and to which the above guidelines refer.

The Instructions can be accessed and downloaded from here: https://www.sfbup.se/wp-content/uploads/2021/02/INS-23749-v.1.0-Instruktion-f%C3%B6r-barn-och-ungdomspsykiatrisk-l%C3%A4kemedelsbehandling-BUP-Stockholm-2021.pdf

i) ***Swedish Association of Child and Adolescent Psychiatry*** guidelines (in Swedish, Svenska Föreningen för Barn- och Ungdompsykiatri, SFBUP), developed by child and adolescent psychiatrists, with the aim of providing guidance to clinicians within child and adolescent psychiatric clinics (BUP).

Clinical guidelines published by the Swedish Association of Child and Adolescent can be found here (from where different guidelines for specific diagnoses can be downloaded)***:***
https://www.sfbup.se/vardprogram/
