# Supplementary material for: Are all children treated equally? Psychiatric care and treatment receipt among migrant, descendant and majority Swedish children: a register-based study
Source: Epidemiol Psychiatr Sci. 2022 Apr 19;31:e20. doi: 10.1017/S2045796022000142 (PMC9069577; doi:10.1017/S2045796022000142)
Supplement: Supplementary file 1 [file S2045796022000142sup001.zip › supplementary material 1 (Icd10 DSM4).pdf]

Supplementary material 1

| **Diagnosis** | **ICD-10 codes** | **DSM-4 codes** | **Diagnosis code number and description in the Child and Adolescent Psychiatry register (BUP register)** |
|---|---|---|---|
| Substance use disorder | F10-F19<br>F55.9 | 291.8<br>292.89<br>292.9<br>303.00<br>303.90<br>304.90<br>305.00<br>305.20<br>305 .90 | 17 Substance abuse<br>(In Swedish: ”Missbruk) |
| Psychotic disorder | F20-F29<br>F05<br>F06.0-2 | 293.81<br>293.82<br>295 (excluding 295.5)<br>297.1-297.3<br>298.8-298.9 | 18 Schizophrenia and other psychotic syndromes”<br>(In Swedish: ”Schizofreni och andra psykotiska syndrom” |
| Bipolar disorder | F30-F31 | 296 | |
| Mood disorder | F32-F34, F38-F39<br>F06.3 | 300.4<br>311<br>296<br>301.13<br>293. 83 | 19 Mood disorders<br>(In Swedish: ”Förstämningssyndrom”) |
| Mild/moderate depression | F320<br>F330 | | |
| Severe depression | F322<br>F323<br>F332<br>F333 | | |
| Anxiety disorder | F41<br>F400<br>F930 | 300.00-02<br>300.21 300.22 | 13 Anxiety disorder<br>(In Swedish: ”Ångeststörning”) |

Supplementary material 1

| | | | |
|---|---|---|---|
| Obsessive compulsive disorder (OCD) and body dysmorphic disorder (BDD) | F42<br>F45.2A | 300.7 | 14 Obsessive-compulsive disorder<br>(In Swedish:"Tvångssyndrom" |
| Tourette syndrome and other tic disorders | F95.0<br>F95.1<br>F95.2<br>F95.8<br>F95.9 | 307.20<br>307.21<br>307.22<br>307.23<br>300.3 | 10 Tic disorder and Tourette syndrome<br>(In Swedish: "Tics, Tourettes syndrom" |
| Post traumatic stress disorder (PTSD) | F431<br>F620 | 309.91 | 15 PTSD |
| Eating disorder | F50 | 307.1<br>307.51<br>307.50 | 23 Eating disorder<br>(In Swedish: Ätstörningar) |
| Sleep disorder | F51 | 307.4 | 24 Sleep disorder<br>(In Swedish: "Sömnstörning") |
| Intellectual disability | F70-F79 | 317<br>318<br>319 | 1 Intellectual disability<br>(In Swedish: "Mental retardation") |
| Autism spectrum disorder | F84 | 299.00<br>299.80 | 5 Pervasive developmental disorders<br>In Swedish: "Genomgripande störning i utvecklingen" |
| Oppositional defiant disorder (ODD) and conduct disorder (CD) | F913<br>F918<br>F919 | 313.81<br>312.8<br>312.9 | 7 Conduct disorder<br>(In Swedish: "Uppförandestörning")<br>8 ODD<br>In Swedish: "Trotssyndrom, utagerande stört beteende UNS" |
| ADHD | F90 | 314.00<br>314.01 | 6 Hyper activity disorder with attention deficit |

Supplementary material 1

| | | | |
|---|---|---|---|
| | | 314.9 | In Swedish: ”Hyperaktivitetssyndrom med uppmärksamhetsstörning”) |
| Neurodevelopmental disorder | F70-F79<br>F84<br>F90 | 299<br>314<br>317<br>318<br>319 | 1 Intellectual disablity<br>5 Pervasive developmental disorders<br>6 Hyper activity disorder with attention deficit |
| Selfharm and injury with unclear intent | X60-X84<br>Y10-Y34 | | 26 Suicide attempt<br>(In Swedish: ”Självmordsförsök”) |
