# Supplementary figures and images for: Are all children treated equally? Psychiatric care and treatment receipt among migrant, descendant and majority Swedish children: a register-based study
Source: Epidemiol Psychiatr Sci. 2022 Apr 19;31:e20. doi: 10.1017/S2045796022000142 (PMC9069577; doi:10.1017/S2045796022000142)

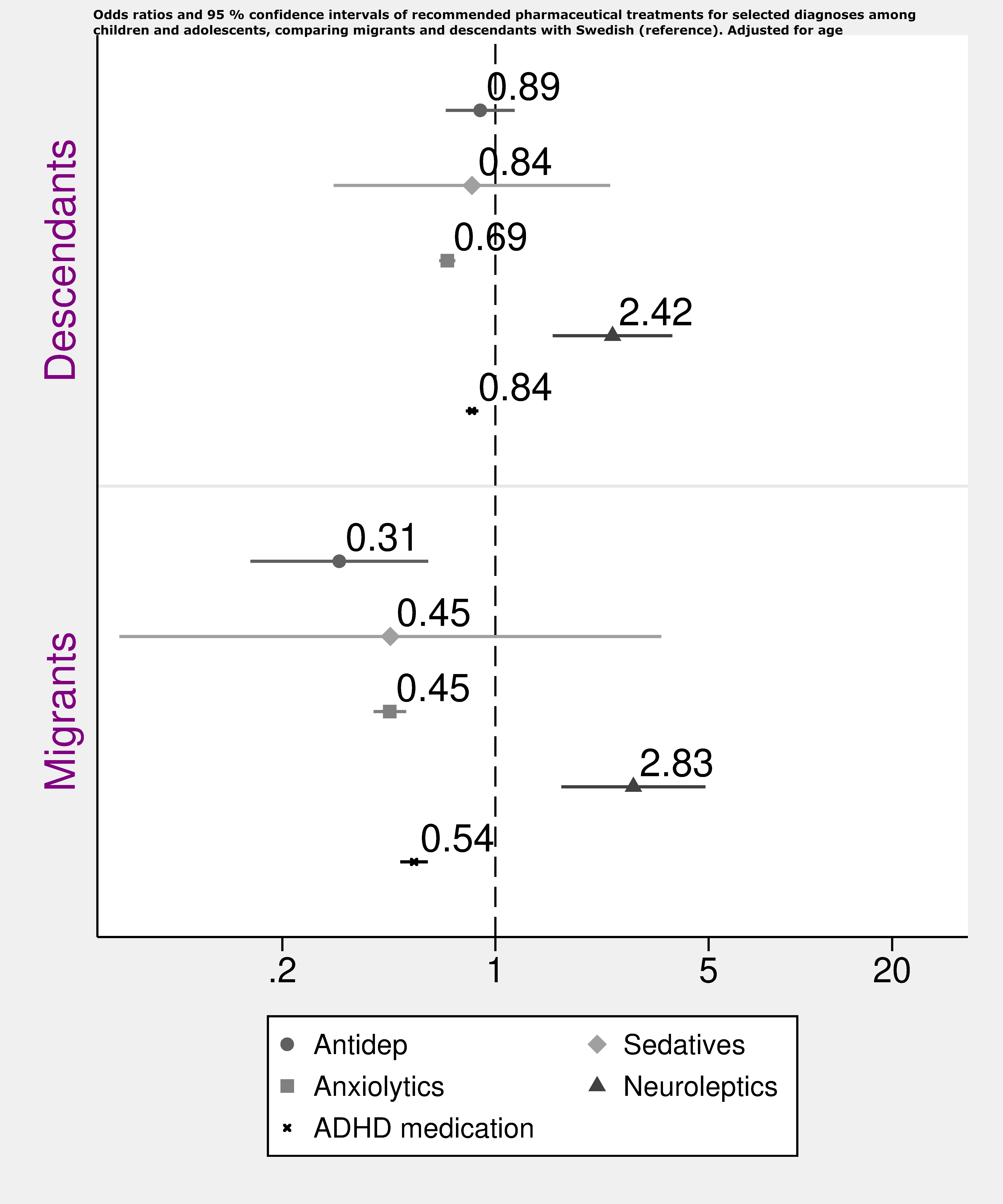

Supplement: Supplementary file 1 [file S2045796022000142sup001.zip › Supplementary_material_5_revised.tif]

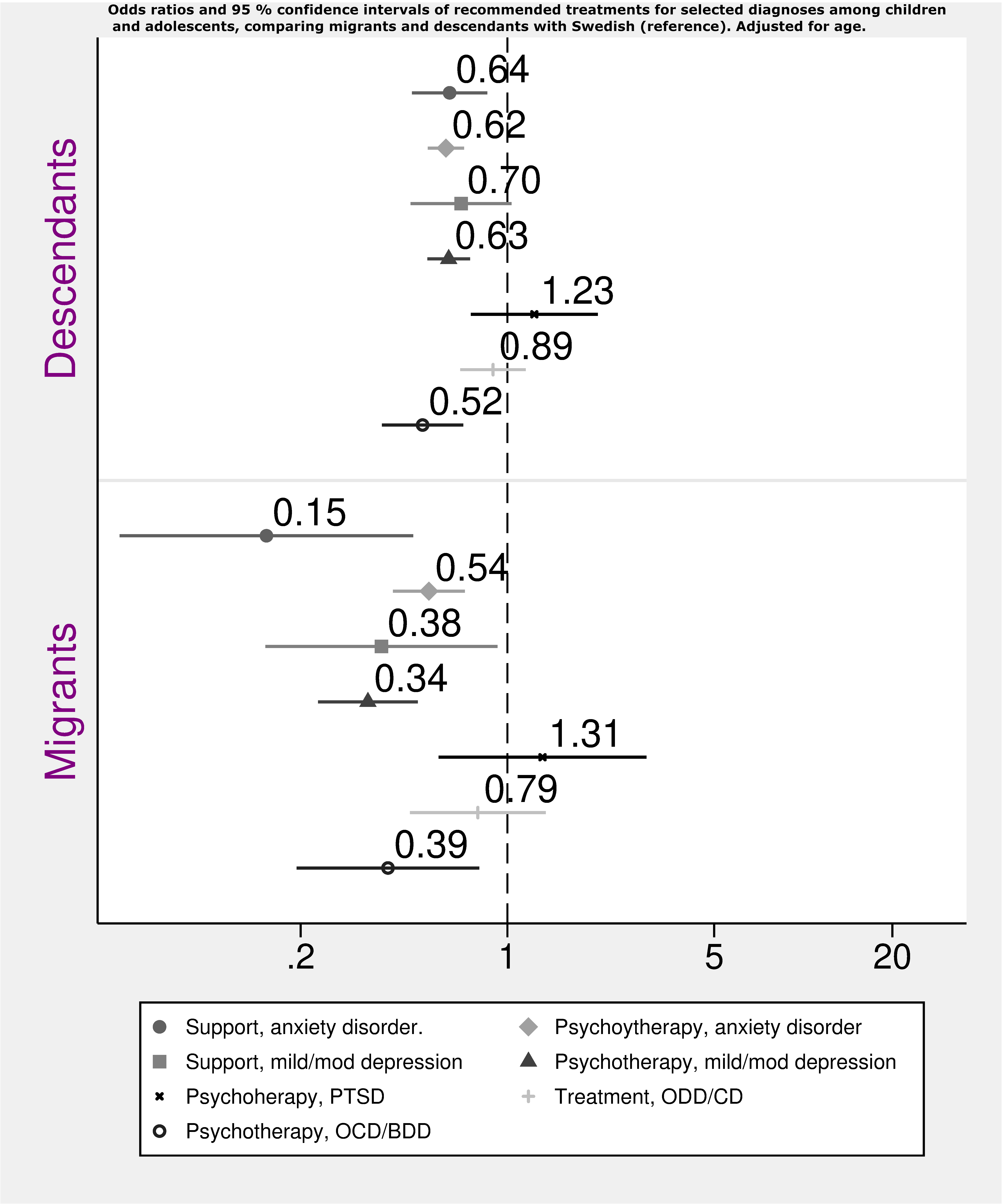

Supplement: Supplementary file 1 [file S2045796022000142sup001.zip › Supplementary_material_4_revised.tif]

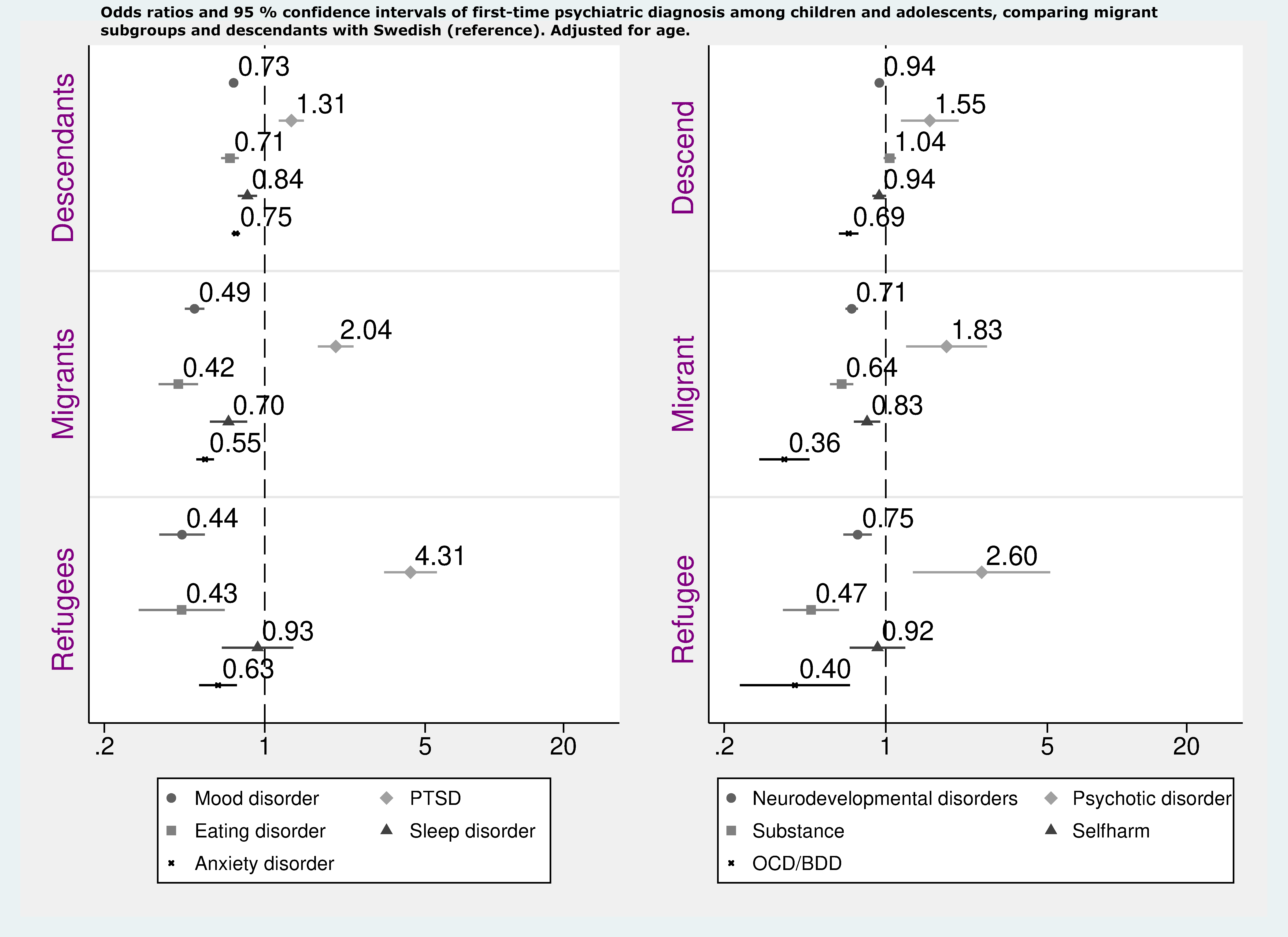

Supplement: Supplementary file 1 [file S2045796022000142sup001.zip › Supplementary_material_3_revised.tif]
